# Colorectal Cancer Diagnosed During Pregnancy With Delayed Treatment

**DOI:** 10.7759/cureus.8261

**Published:** 2020-05-24

**Authors:** Suzanne Cao, C. Camille Okekpe, Inessa Dombrovsky, Guillermo J Valenzuela, Kristina Roloff

**Affiliations:** 1 Department of Women's Health, Arrowhead Regional Medical Center, Colton, USA; 2 Obstetrics and Gynecology, Arrowhead Regional Medical Center, Colton, USA

**Keywords:** colorectal cancer, high-risk pregnancy, treatment choices, treatment dilemma

## Abstract

Colorectal cancer during pregnancy is rare. Because of a pattern of delay in childbearing and because colorectal cancer is now diagnosed more often in young adults, the incidence is expected to rise. Diagnosis during pregnancy is challenging as many of the symptoms mimic common pregnancy symptoms. Colonoscopy is the gold standard for diagnosis, but pregnancy is a relative contraindication to colonoscopy. Once diagnosed, pregnant women often have more advanced disease. Due to its rarity, treatment is often based on case reports and limited studies. A multidisciplinary team is important in the optimization of treatment.

We present a case of a 29-year-old African-American primigravid with chronic gastrointestinal symptoms diagnosed with colorectal adenocarcinoma at 17 weeks of gestation. She delayed surgical intervention for several weeks due to fear of miscarriage, and ultimately underwent exploratory laparotomy with hemicolectomy and colostomy placement at 20 weeks. Abdominal ultrasound and magnetic resonance imaging revealed non-specific hepatic lesions concerning for metastatic disease, but the patient refused biopsy due to concern for radiation harm to the fetus. Chemotherapy was considered, but postponed until the postpartum period, for fear of fetal harm. Computed tomography imaging after delivery noted an increased number of hepatic lesions, representing progression of her disease. She received two rounds of chemotherapy in the postpartum period, but remained non-compliant with treatment recommendations and ultimately was lost to follow-up.

This case presents a delayed diagnosis of colorectal cancer in pregnancy, as well as delayed treatment due to concerns for fetal harm with current therapies. It emphasizes the diagnostic challenges and the complexity and ethical issues involved when a pregnant patient faces a life-threatening terminal illness. This case adds to the growing body of literature on colorectal cancer in pregnancy and highlights the importance of clinical suspicion, informed patient centered decision making, and tailored treatment goals.

## Introduction

Colorectal cancer (CRC) during pregnancy is rare, with an incidence ranging from 0.0008% to 0.008% [1-4]. The most common cancers diagnosed during pregnancy are breast, cervical, and hematologic cancers, which have a peak incidence in the reproductive years [5]. Trends in the United States and Europe are towards later childbearing [6]. CRC is now being diagnosed in younger women, and so it follows that the incidence of CRC in pregnancy will likely rise [7-10]. Bailey et al. analyzed the Surveillance, Epidemiology, and End Results database and demonstrated an approximate annual increase in CRC incidence rate of 2% in 20-34 year olds [9]. By 2030, the incidence of CRC in this age group is predicted to increase by 90%-124% [9].

The diagnosis of CRC during pregnancy is extremely complicated and challenging. Symptoms of CRC often mimic common pregnancy symptoms, such as abdominal pain, constipation, anemia, fatigue, rectal bleeding, nausea, vomiting, and weight loss [11]. This often delays the diagnosis, especially if the index of suspicion is not high [12]. As a result of delayed diagnosis, complications such as obstruction, perforation, and metastatic spread may be found more frequently in pregnant patients, which may lead to poorer prognosis [13]. Additionally, imaging and procedures may be delayed for fear of damage to the fetus. Colonoscopy is the gold standard to diagnose CRC. However, pregnancy is a relative contraindication due to possible complications including fetal exposure to medication, utero-placental insufficiency due to maternal hypoxia or hypotension, and placental abruption due to mechanical pressure [14-16]. Compared to the general population, 86% of tumors in pregnant women occur below the peritoneal reflection [17]. These tumors may be diagnosed using flexible sigmoidoscopy without sedation or radiation and avoids the risk of colonoscopy [17]. Carcinoembryonic antigen (CEA) during pregnancy may be elevated in the presence of a CRC, but usually is within the normal range, and thus is not a good screening tool for CRC in pregnancy [14,15,18].

A comparison of pregnant and non-pregnant women controlled for stage of CRC shows a similar five-year survival rate [15,18]. However, pregnant women often have more advanced disease at the time of diagnosis. Diagnosis and treatment should involve a multidisciplinary team including the obstetrician, maternal fetal medicine (MFM) physician, colorectal surgeon, and oncologist. Given the rarity of CRC in pregnancy, treatment is often based on case reports and limited studies. Treatment must take into account the estimated gestational age of the fetus, elective or emergent clinical presentation, location and stage of the cancer, and the patient’s wishes [19]. Optimization of treatment must weigh the risks and benefits to both the mom and the unborn fetus. It is vital to remember that the patient’s needs and beliefs are intimately involved in the decision-making process.

We present a case of a young African-American woman diagnosed with CRC at 17 weeks of pregnancy. Her chronic gastrointestinal symptoms were initially attributed to irritable bowel disease, and led to a delay in diagnosis. Once her CRC was diagnosed, she elected to delay her treatment for the benefit of her unborn fetus knowing the risk to her own health. The patient’s informed consent for publication of this report was obtained.

## Case presentation

A 29-year-old African-American primigravid presented for consultation with MFM for severe anemia at an estimated gestational age of 17 weeks and three days dated by a nine-week ultrasound. Her past medical history was significant for chronic rectal bleeding with passage of tissue for several years. She had been experiencing bright red blood per rectum since the age of 17 years, which she thought was due to hemorrhoids. Approximately one year prior to conception, she started to experience severe abdominal pain with a change in her bowel movements. She described her bowel movements as soft, greasy, and red-streaked with occasional blood clots and pink puffy tissue. She presented to various local emergency rooms for these symptoms, but was never worked up with a diagnostic procedure or given a formal diagnosis. Four weeks prior to her MFM consultation, she was admitted to another hospital for severe abdominal pain, rectal bleeding, and fatigue. She was diagnosed with colitis and received one unit pack red blood cell transfusion and intravenous iron for a hemoglobin (Hb) of 6.6 g/dL and a hematocrit (Hct) of 21.5%. She was instructed to continue weekly intravenous iron, but was non-compliant. Following her discharge, she complained of continued fatigue, weakness, and occasional dizziness with continued rectal bleeding and passage of tissue, with approximately 9-10 episodes a day. The patient also noted a weight loss of approximately 20 lbs prior to pregnancy, and an additional 10 lbs since finding out she was pregnant, but attributed it to hyperemesis gravidarum.

Her medications included prenatal vitamins, mesalamine 800 mg TID for the past two months, pantoprazole 40 mg daily, and Tylenol as needed. She endorsed a history of smoking and snorting methamphetamine for two years, but stated last use was six to seven months prior. She was formally a heavy alcohol drinker consisting of 12 beers a day for the last six years, but quit eight months prior. She had a family history of breast cancer, colon cancer, and lung cancer in multiple family members.

A fetal anatomy ultrasound demonstrated a singleton gestation without evidence of anomalies. The patient’s vitals were normal and stable, and she was noted to be 114 lbs, 5’2” with a body mass index of 21 kg/m^2^. Her stated pre-pregnancy weight was 125 lbs. Rectal exam revealed no evidence of hemorrhoids or rectal masses and fecal occult blood testing was negative. She was admitted for further workup of the severe anemia and possible active colitis.

**Figure 1:**
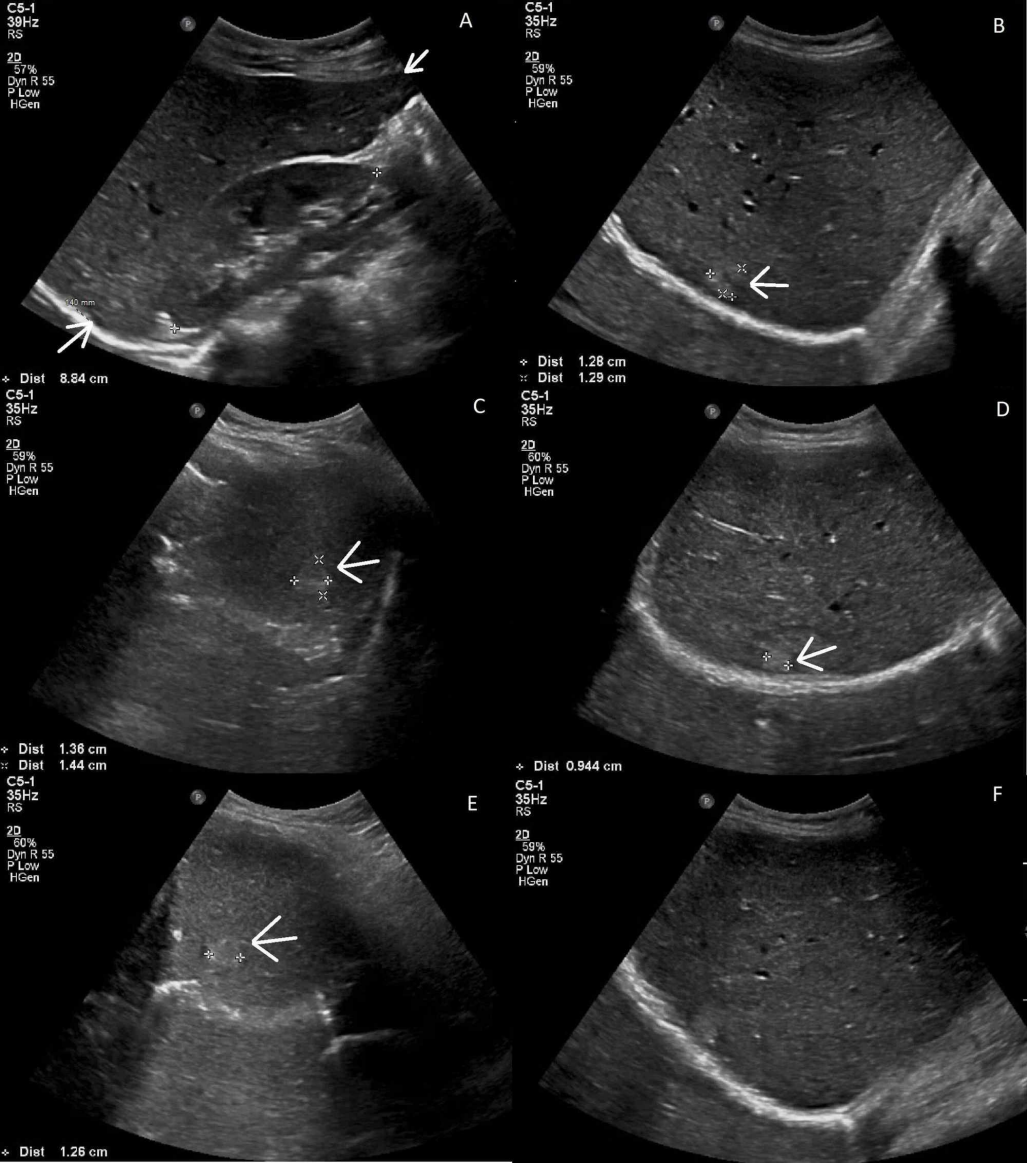
Abdominal ultrasound imaging (A) Two non-specific hepatic lesions. (B, C) 1.4 cm lesion. (D, E) 1.2 cm lesion. (F) Normal liver.

Laboratory results demonstrated an Hb 8.4 g/dL, Hct 27.0%, mean corpuscular volume 79 fL, negative fecal occult blood test, reticulocyte count 1.8%, erythrocyte sedimentation rate 62 mm/hr, C-reactive protein 3.92 mg/dL, iron 26 µg/dL, ferritin 28.2 ng/mL, and normal liver function tests. A gastroenterologist consultation led to a flexible sigmoidoscopy, which showed a large, near obstructing friable mass 15 cm from the anal verge. Biopsy results demonstrated invasive colonic adenocarcinoma. CEA was 8.6 ng/mL. Abdominal ultrasound demonstrated two hyperechoic hepatic lesions measuring 1.4 and 1.2 cm (Figure 1).

**Figure 2:**
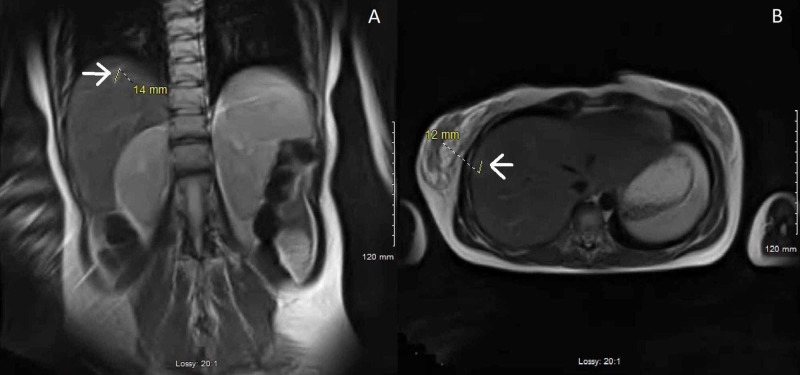
Magnetic resonance imaging (A, B) Two non-specific hepatic lesions.

Magnetic resonance imaging demonstrated two non-specific liver lesions and wall thickening of the rectum (Figure 2).

A triple phase computed tomography (CT) imaging scan was recommended to aid in staging, but the patient declined due to concern for radiation exposure to the fetus.

A multidisciplinary team, including MFM, obstetrics, colorectal surgery, and oncology, was formed to coordinate her care plan. She was offered termination of pregnancy given the new diagnosis of CRC, which she refused. Due to the size and location of the mass, there was a concern about progression to obstruction without intervention, and surgery was recommended. The patient initially desired delaying any intervention until after she delivered given the possible risks of miscarriage. However, she was extensively counseled that without surgery, the tumor would enlarge and cause bowel obstruction, necessitating emergent intervention and increased harm to herself and her unborn fetus. The patient continued to refuse immediate surgical intervention until three weeks later. The patient stated that although still fearful of fetal loss, she would undergo surgery to prevent possible future emergent intervention.

She underwent an exploratory laparotomy at 20 weeks of gestation with a low anterior resection with a total mesorectal excision, end colostomy, and para-aortic and paracaval node resection. The rectum was poorly visualized behind the gravid uterus. Indomethacin was given for 48 hours following surgery to prevent preterm contractions. Pathology demonstrated a moderately differentiated colonic adenocarcinoma with necrosis and invasion through the muscularis propria into the pericolorectal tissue. Thirteen of the 22 lymph nodes sampled were positive, including the para-aortic ones. Her final diagnosis was stage III rectosigmoid adenocarcinoma (pT3pN2bM0). She refused further workup of her non-specific liver lesions, which if positive for metastatic disease would have been stage IV.

The patient’s pregnancy was followed with serial growth ultrasounds every four weeks demonstrating normal interval growth and she was started on non-stress testing at 30 weeks of gestation. She received genetic counseling given her family history and young age of diagnosis of CRC. Her BRCA gene mutation tests were negative. She was heterozygous for a valine to leucine substitution on amino acid position 32 on the second exon on the PMS2 gene. Given limited information about this mutation, it was classified as a variation of unknown significance. The remaining genetic workup was negative. Immunohistochemical expression of MLH1, MSH2, MSH6, and PMS2 was detected on the rectal biopsies and therefore not consistent with Lynch syndrome or hereditary non-polyposis colorectal cancer.

Chemotherapy and radiation are normally indicated following surgical resection of stage III CRC. Because of her pregnancy, radiation was contraindicated. She was offered chemotherapy and explained the unknown long-term risk on the fetus. Few case reports suggested that the chemotherapy regimen may increase her chances of preterm delivery and fetal growth restriction [20]. In addition, she was counseled that chemotherapy without radiation for stage III CRC may or may not improve her long-term prognosis or survival. She ultimately elected to forgo any additional treatment during her pregnancy due to concern for fetal well-being.

**Figure 3:**
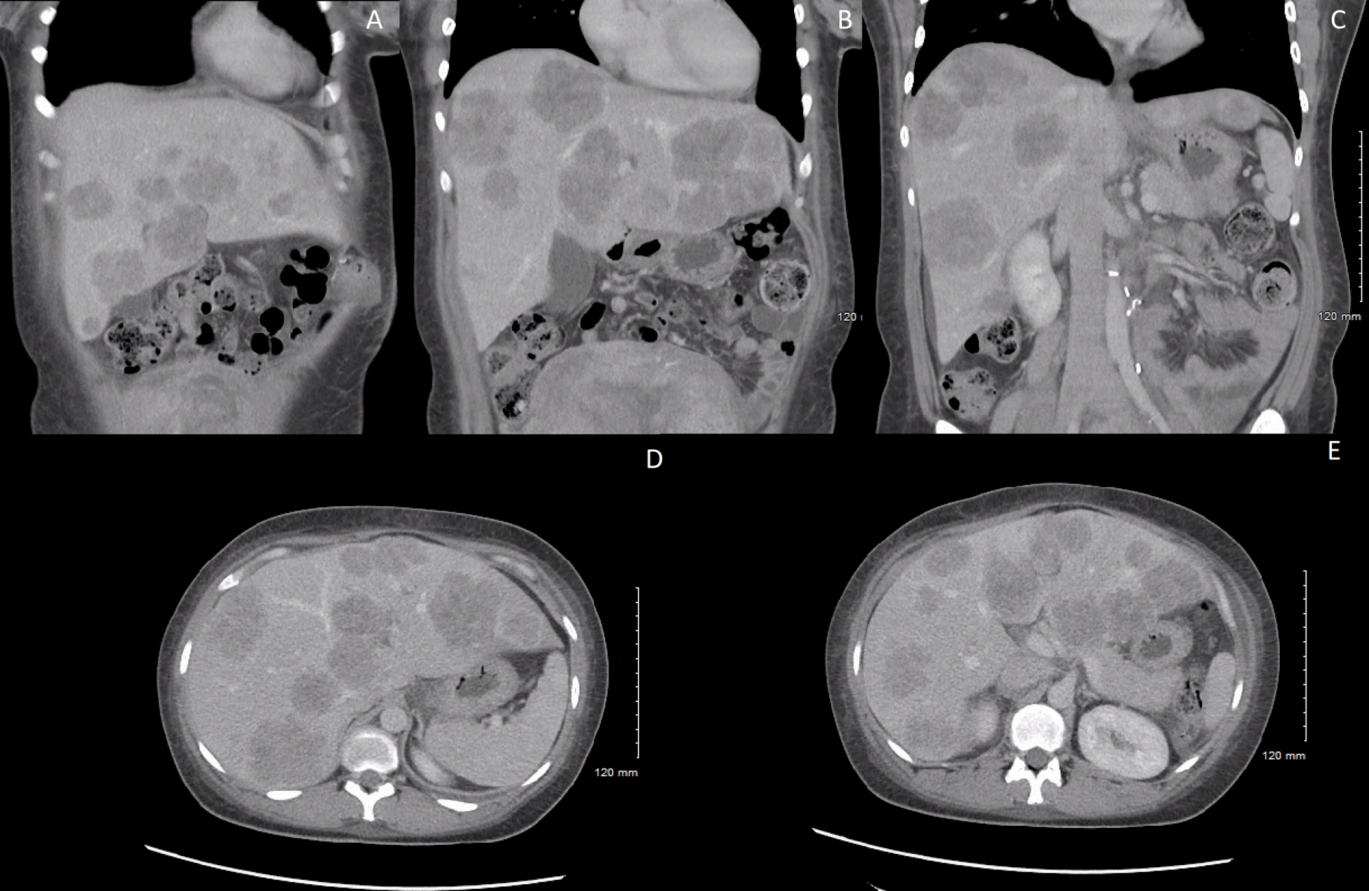
Computed tomography imaging (A-E) Multiple diffuse hepatic lesions representing metastasis

The team recommended delivery at 34 weeks of pregnancy after weighing the risk of preterm birth for the fetus and the benefit of initiating radiation and chemotherapy treatment sooner for the mother to improve her prognosis. The patient underwent induction of labor and had an uncomplicated vaginal delivery of a male infant, weighing 2,620 g with Apgar scores of 8 and 9 at one and five minutes, respectively. The newborn was noted to have hypospadias and a hooded foreskin and chordee, but otherwise grossly normal physical exam, laboratory results, and vital signs. He was discharged home on day 2 of life. After delivery, a CT scan was obtained demonstrating multiple diffuse liver masses, at least 20, concerning for metastasis (Figure 3).

The repeat CEA was 187.7 ng/mL. Given confirmation of stage IV CRC, the oncologist recommended treatment with FOLFOX (fluorouracil/5-FU and oxaliplatin) and avastin. The patient completed two cycles of chemotherapy before being lost to follow-up.

## Discussion

Our patient had chronic gastrointestinal symptoms, but never underwent diagnostic colonoscopy or flexible sigmoidoscopy prior to pregnancy. Her symptoms of severe anemia and rectal bleeding were attributed to irritable bowel disease and hemorrhoids, and her early pregnancy weight loss was attributed to hyperemesis gravidarum. Despite seeing multiple providers and the patient’s symptoms, there were missed opportunities to diagnose CRC earlier. As the incidence of CRC in young adults is expected to rise, primary care providers have the ability to make the diagnosis early to expedite treatment and hopefully improve outcomes. Therefore, it is imperative that primary care providers take a careful history, perform a thorough physical exam, and have a high index of suspicion for CRC when young patients present with persistent or worrisome symptoms.

This case demonstrates the importance of intimately involving the patient in the decision-making process. Surgery, chemotherapy, and imaging of suspicious hepatic lesions were delayed at the discretion of our patient for fear of fetal harm, with the knowledge of possible sacrifice of her own health. Ultimately, it is the informed patient who decides the balance of treatment risk to her unborn fetus for the benefit to herself. The team must be willing to adapt treatment plans according to the patient’s wishes. We also must be cognizant of our ability to support her decision, even when she chooses a treatment course that may differ from our own personal beliefs and desires. Physicians should be sensitive and understanding regarding the complexity and ethical issues surrounding pregnant patients with a possible life-threatening terminal illness. The prognosis of CRC in pregnancy is largely unknown, and the ideal treatment plan has not been established. As the incidence and the body of literature grows, it may lead to more substantiated recommendations. 

## Conclusions

Here we present a case of delayed diagnosis of CRC in pregnancy, as well as delayed treatment of care based on patient preference. The case emphasizes the diagnostic challenges and the complexity and ethical issues involved when a pregnant patient faces a life-threatening terminal illness. While medical treatment plans involve a calculated balance of risks and benefits, the ultimate decision to proceed with any treatment during pregnancy lies with the patient. This case adds to the growing body of literature on CRC in pregnancy and highlights the importance of clinical suspicion for the diagnosis, as well as the need for coordinated multidisciplinary team efforts towards informed patient centered decision making and tailored treatment goals.
